# Transcriptome and Metabolome Analysis of Upland Cotton (*Gossypium hirsutum*) Seed Pretreatment with MgSO_4_ in Response to Salinity Stress

**DOI:** 10.3390/life12060921

**Published:** 2022-06-20

**Authors:** Wei Ren, Li Chen, Qian Wang, Yanping Ren

**Affiliations:** 1 State Key Laboratory of Desert and Oasis Ecology, Xinjiang Institute of Ecology and Geography, Chinese Academy of Sciences, Urumqi 830011, China; renwei@ms.xjb.ac.cn (W.R.); wangqian@ms.xjb.ac.cn (Q.W.); 2 Fukang Station of Desert Ecology, Chinese Academy of Sciences, Fukang 831505, China; 3 College of Agriculture, Xinjiang Agricultural University, Urumqi 830052, China; ryp923@xjau.edu.cn

**Keywords:** RNA-seq, salinity tolerance, seed treatment, cotton, DEGs, DAMs

## Abstract

Upland cotton (*Gossypium hirsutum*) is a salt-tolerant crop that can withstand high salinity levels without showing signs of harm to the plant. However, the plant is more prone to salinity stress at the germination stage and a poor germination as well as poor crop stand lead to a weak productivity. It is possible to obtain a comprehensive picture of the cotton seedling germination and establishment against salt stress by examining dynamic changes in the transcriptomic and metabolomic profiles. The reported study employed a pretreatment of cotton seeds by soaking them in 0.2% Magnesium Sulphate (MgSO_4_) solution at room temperature for 4, 8, and 12 h. The analysis of variance based on the studied traits emergence rate, above and underground plant parts’ fresh weight measured, displayed significant differences of the three treatments compared with the control. A total of 28,801 and 264 differentially expressed genes (DEGs) and differentially accumulated metabolites (DAMs) were discovered to code for biological processes such as response to salt stress, cellular response to salt stress, abscisic acid receptor PYR/PYL, regulation of seed growth and germination, and auxin-activated signaling pathways. A large amount of ethylene-responsive transcription factors (ERF) was identified (1235) as differentially expressed, followed by *bHLH* (252), *WRKY* (96), *MYB* (202), *GATA* (81), *RABA* (64), *DIVARICATA* (28), and *MADs*-box (26) in treated seedling samples. Functional enrichment analysis revealed the significant roles in the hormones and signal transduction, carbohydrates metabolism, and biosynthesis of amino acids, promoting salt stress tolerance. Our results indicated positive effects of MgSO_4_ at 4 h treatment on seedling germination and growth, seemingly by activating certain growth-regulating enzymes (auxins, gibberellins, jasmonates, abscisic acid, and salicylic acid) and metabolites (phenolic acids, flavonoids, and akaloids). Such pretreatment of MgSO_4_ on seeds would be beneficial in future cotton management under saline conditions to enhance good crop stand and productivity.

## 1. Introduction

Germination to break seed dormancy is an essential physiological process in the life cycle of plants as it determines the success or collapse of future plant growth and establishment [1]. This process is sensitive to adverse climatic fluctuations, particularly drought, salinity, and temperature [2,3]. Almost 7% of the arable land across the globe is saline and this proportion is increasing due to many reasons, particularly irrigation with contaminated or low-quality water and poor drainage [4,5]. If proper management and sustainable agriculture are not carried out, this proportion could rise up to 50% by 2050 [6]. Approximately 90% of the food crops are salt sensitive (glycophytes) and suffer from significant yield losses even under moderate salinity conditions [7,8].

Soil salinity is the concentration of all soluble salts in the soil water measured as electrical conductivity (EC), represented in unit dS m^−1^ [9]. The classification of saline soil types depends on the concentration of the type of salt present, such as sodic, saline, and sodic-saline, or the proportion of Na^+^, Mg^2+^, or Ca^2+^ in it [10,11]. The dilemma with saline soil is the existence of soluble salts in it, majorly SO_4_^2−^, NO_3_^−^, and Cl^−^. Generally, saline soils have a pH below 8.5 and sodic soils bear a pH up to 10. Salinity disturbs germination by creating osmotic and oxidative stress, resulting in less germination rate with prolonged germination time [12]. When the salt concentration around roots is greater, osmotic potential around roots is created and water uptake will be reduced [13]. The osmotic and ionic effects initiate the production of reactive oxygen species (ROS) and thus oxidative damage, leading to the disruption of lipids, proteins, nucleic acid, and other organellar structures [12].

Different methodologies have been employed for enhancing plant tolerance against different stresses under stressful environments; some are particularly time-consuming (e.g., conventional breeding), and others are currently unacceptable in many countries around the world (e.g., plant genetic modifications). Seed pretreatment is a rapidly emerging field in plant stress physiology [14]. Plants treated with certain natural or synthetic compounds and/or biological agents before stress events show enhanced tolerance when exposed to sub-optimal abiotic conditions. Seed pretreatments have become very popular in recent decades [15]. Recently, seed pretreatments with a low dose of salts, antioxidants, trace elements, amino acids, phytohormones, and other signaling molecules were found to provide an enhanced stress tolerance under stressful conditions [2]. Stress impacts on plant growth and yield in pre-treated plants are remarkably reduced compared with non-treated plants. In cotton (*Gossypium hirsutum*), germination/emergence and seedling development are highly prone to salinity [16]. The effect of seed pretreatment must be mainly studied during germination [17].

It is assumed that the cotton plants respond to salt stress by maintaining K^+^ and Na^+^ ions balance across their tissues; maintaining a higher K^+^/Na^+^ ratio across the tissues is more critical than simply maintaining the lower concentration of Na^+^ ions [18,19,20]. Moreover, Mg and Ca are also considered important in improving cotton salt tolerance [21]; however, it has previously been reported that Mg^+2^ and Ca^+2^ might not reduce the toxicity of Na^+^ in cotton at the seedling stage [22]. Due to salinity in the growing mediums, the plants suffer from followings such as decreased photosynthetic activity and carbon assimilation, lesser stomatal density and conductance, and increased mesophyll resistance, thus reducing efficiency of light by PS-I and II [23]. Although many studies investigating the response of cotton to salt stress and the improvement of salt tolerance by mineral elements have been conducted [24,25], most of these studies have focused on the effects of salt stress on one or several mineral elements and the responses to other elements while their dynamic changes to salt stress have not been fully elucidated [26].

The prominent adverse effects of salinity in plants include osmotic and oxidative stress as well as ion toxicity. The plant responds to osmotic stress via osmotic adjustments such as cellular productions of soluble sugars, betaine, proline, glycine, and several other osmolytes [27]. For example, proline is a low-molecular-weight significant substance for osmotic adjustment found in the free state with no net charge and high-water solubility. Such types of osmolytes become activated and accumulated in the cell for osmotic adjustments to alleviate salt stress [27,28]. Salt-stress condition during the germination process induces production of endogenous phytohormones, i.e., salicylic acid (SA) and cytokinins (CYTs) as growth regulators to combat the situation [29]. SA has significant participation in physiological mechanisms under abiotic as well as biotic stresses. CYTs regulate various developmental processes like mitotic cell divisions, vascular and shoots differentiation, nutrient mobilization, senescence of leaves, production of anthocyanins, as well as photosynthetic developments etc. [30,31].

There is a need to explore available cotton to estimate sensitivity or tolerance towards salinity in the growing medium. Gene expressions mainly depend on the regulatory sequences, i.e., transcription factors (TFs). Various genes have been reported earlier in cotton to control their growing medium’s genotype response to salinity levels [32]. For example, mitogen-activated protein kinase kinase (*MKK*) [33], mitogen-activated protein kinase (*MPK*) [34], ethylene-responsive transcription factor (*ERF*) [35], N-terminal and C-terminus DNA binding protein (*NAC*) [36], zinc finger protein (ZFP) [37], 64-amino acid type 3 metallothionein protein (*GhMT3a*) [38], and dehydration-responsive element-binding protein (*DREB*) [39]. A prominent example is transgenic cotton with simultaneous expression of genes TsVP and AtNHX_1_, resulting in higher seed cotton yields grown in salt-affected soil [40].

Many studies documented the salt tolerance mechanism in adult cotton leaves and root tissues with the exploration of specific salt-tolerant genes, but there is a scarcity of research on the effects of pretreatment with different types of salt compounds on the germination rate and establishment of cotton seedlings. Therefore, this experiment utilized a 0.2% MgSO_4_ solution to soak cotton seeds for 0, 4, 8, and 12 h to improve germination and emergence in saline soils to observe which soaking time is the best. Simultaneously, the related mechanisms were explored by looking at corresponding enzymes, proteins, and metabolites encoded and regulated by certain genes and transcription factors through transcriptomic and metabolomic profiling approaches. The study would be significant for cotton production in saline–alkali soils in arid regions of Xinjiang, for example, especially by understanding the role of salinity tolerant genes via their regulation of expression with salt pretreatment.

## 2. Materials and Methods

### 2.1. Experiment Design and Sample Collection

The experiment was conducted at the experimental station of the State Key Laboratory of Desert and Oasis Ecology, Xinjiang Institute of Ecology and Geography (24°28′08″ N; 113°49′49″ E) in pots at the greenhouse in mid-September 2021. The soil used for experimentation was collected from the wasteland of the experimental farm at Xinjiang Agricultural University. Soil texture was alluvial with a gray color and the physio-chemical properties were as follows: salt content (14.63 mgg^−1^), electrical conductivity (EC) (4088 µScm^−1^), Ca^2+^ (1.776 mgg^−1^), K^+^ (0.265 mgg^−1^), Mg^2+^ (0.182 mgg^−1^), Cl^−^ (0.537 mgg^−1^), Na^+^ (2.473 mgg^−1^), SO_4_^2−^ (8.49 mgg^−1^) and pH = 8.03. Before the sowing of cotton seeds, pretreatment was conducted by soaking them in 0.20% MgSO_4_ solution at room temperature for four different treatment periods, i.e., 0 h (CK), 4 h (M4), 8 h (M8), and 12 (M12) h, with three replicates per treatment. On completion of time durations, every soaked sample seed was rinsed with clean water for about 15 min. Ten selected seeds were planted using a metallic cylinder with 13 cm height and 8.5 cm internal diameter inside triplicated pots per treatment with 350 g of soil. Approximately 170 mL of water was poured into each pot. The soil was gently pressed to ensure full contact of seeds with the soil. The seeds were covered with 0.2 cm of surface soil.

### 2.2. RNA Isolation and Transcriptomics

Twelve independent sample seedlings in triplicates were selected to obtain sample tissues from roots and leaves from all three salinity treatments and stored at −80 °C after placement in liquid nitrogen. Preparation and sequencing of 12 mRNA libraries on HiSeq 2000 (Illumina) were outsourced to Qinghai Keju Biotechnology Co., Ltd. (Qinghai, China) after extraction of total RNA from above and underground plant tissues using TRIzol reagent. The main steps included purification of RNA samples, double-stranded cDNA synthesis, joint-addition, and DNA library amplification cum quality detection, and a few more steps followed by transcriptome sequencing. Then, the quality of raw sequenced reads was improved by deserializing them and removing low-quality ones, then reassembling the transcriptome data utilizing the software Velvet/Oases. Furthermore, the obtained cleaned reads’ Q20, Q30, and guanine–cytosine (GC) contents were calculated.

After sequencing, the raw reads obtained were filtered to obtain clean and high-quality reads by eliminating low-quality bases (Q-value ≤ 20), poly-N (>10%) and adaptors, with the help of ‘fastp’ (v 0.18.0) [41]. Simultaneously, GC contents, Q20, and duplication level of sequence in clean data were assessed. These obtained clean data with high quality were utilized for further downstream analyses. The HISAT2 (v 2.4) software [42] was operated to map clean reads of each sample with the reference sequence (http://ccb.jhu.edu/software/tophat/index.shtml, accessed on 3 November 2021). The StringTie (v 1.3.1) tool [43,44] was utilized to assemble the mapped clean reads using each sample’s reference-based approach. This software also provided the FPKM (fragment/kb of transcript/million mapped reads) values for each transcription region to estimate their variation and abundance of expression. The threshold value of false discovery rate (FDR) for the significance of observed differential gene/transcript expression was kept ≤ 0.05 with the absolute value of fold change ≥ 2.

The differential expression of RNAs between two groups of samples was assessed with the help of the DESeq2 tool [45]. Gene Ontology (GO) [46] of mapped DEGs in the database (http://www.geneontology.org/, accessed on 3 November 2021) was accomplished by calculating their gene numbers regarding each GO term, followed by the hypergeometric test. It involved the evaluation of significant enriched GO terms related to DEGs while comparing the whole transcriptome background. Gene function annotation was conducted on databases, i.e., GO and KO (KEGG Ortholog database) (http://www.genome.jp/kegg/, accessed on 3 November 2021). The Blastall tool was utilized further to identify and annotate the significant enriched metabolic pathways related to the DEGs on the KEGG database [47]. Furthermore, gene set enrichment analysis was carried out by GESA and MSigDB software [48] to determine the significant differences among genes regarding GO terms and pathways.

### 2.3. Network Analysis

The identified DEGs were further scrutinized while investigating their protein–protein interactions with the help of String v10 [49]. It gave networks of hub-genes harboring nodes and lines to reveal genes and interactions among them, respectively. The resultant files comprising these networks were visualized with the help of Cytoscape (v3.7.1) software [50].

### 2.4. Sample Extraction and Metabolome Profiling

The sample preparation for the extraction and quantification of metabolites was performed by Norminkoda Biotechnology Co., Ltd. (Wuhan, China) [51]. An amount of 100 mg of vacuum freeze-dried cotton tissues (above and underground plant parts) fine powder was dissolved in 1.0 mL methanol (70%) by vortex for 30 min for 30 s each time and kept at 4 °C overnight. Then, after centrifugation at 12,000 rpm for 10 min, extracts were filtered (0.22 µm pore size) and analyzed via UPLC-MS/MS system (UPLC, SHIMADZU CBM30A, www.shimadzu.com.cn/, accessed on 13 September 2021; MS/MS), (4500 QTRAP, http://sciex.com/, accessed on 13 September 2021). The qualitative analysis was accomplished based on secondary spectral information. Metabolite quantification was carried out using triple and quadruple mass spectrometry through multi-reaction monitoring (MRM) analysis. LIT and triple quadrupole (QQQ) scans were developed on a triple, quadruple linear ion trap mass spectrometer (Q TRAP). The metabolite data were analyzed via Principal component analysis (PCA), orthogonal partial least squares discrimination analysis (OPLS-DA), cluster analysis, and Pearson’s correlation analysis using R software package MetaboAnalystR [52]. The metabolites identified through them were subjected to the OPLS-DA model [53]; then, the metabolites with fold change >2 or <0.5 and variable importance in projection (VIP) values >1 were taken as differential metabolites for the discrimination of treatments and control groups. Moreover, the KEGG pathway database (http://www.kegg.jp/kegg/pathway.html, accessed on 3 November 2021) [47] was utilized for the classification and pathways enrichment analyses related to differentially accumulated metabolites (DAMs) to determine their related key pathways.

### 2.5. Conjoint Analysis

The systematic and comprehensive integrated statistical analyses of transcriptome and metabolome data for cotton above and undergorund biomass to establish the relationships between biomolecules at various levels were conducted. They were carried out via a combination of biological functional analyses, and correlation analysis, metabolic regulatory pathways and funtion annotation analyses, simultaneously to screen out key genes or metabolic regulatory pathways involved in the salinity tolerance mechanisms. The genes related to secondary metabolites biosynthesis and metabolic pathways were selected for analysis. The batch data after normalization were used for the analysis via R software in ‘cor’ package. Pearson’s correlation coefficient R^2^ ≥ 0.8 with *p*-values  ≤  0.05 was utilized for the correlation analysis and corrected for Bonferroni multiple test. Cytoscape software was utilized to extract the relationship between transcriptome and metabolome data.

### 2.6. qRT-PCR Verification

The RNA tissue samples from MgSO_4_-treated seedlings after 4, 8, and 12 h were collected to examine and verify through quantitative real-time PCR. Total RNA was extracted from above and underground plant parts using TRIzol reagent (Invitrogen) following the manufacturer’s protocol. Complementary DNA was synthesized using a PrimeScript RT reagent kit with gDNA eraser (TaKaRa). Cotton *Actin9* (*GhActin9*) was selected for normalization. Primers were designed in Primer Premier 5.0 (Premier Biosoft International, Palo Alto, CA, United States). Each 50 μL reaction sample was run on a Bio-Rad IQ2 sequence detection system with Applied Biosystems software. Relative expression was calculated using the 2^−ΔΔCt^ method.

## 3. Results

**Figure 1:**
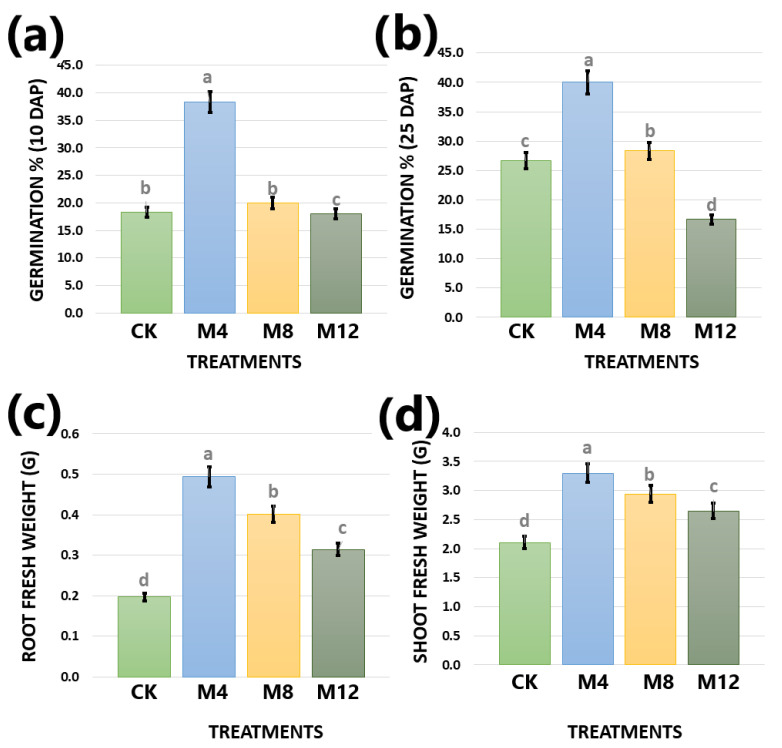
Mean comparisons of phenotypic traits at different MgSO_4_ treatments on cotton seed under study; (**a**) germination percentage at 10 days after planting, (**b**) germination percentage at 25 days after planting, (**c**) root fresh weight at 25 days after planting (**d**) shoot fresh weight at 25 days after planting. Plots showed statistical differences among treated samples. Bar plots with overlapping error bars are statistically insignificant, similarly, letters on bars show statistical significance if samples do not share these letters with each other and vice versa. CK: control (0 h), M4: 4 h treatment of seeds with MgSO_4_, M8: 8 h treatment of seeds with MgSO_4_, M12: 12 h treatment of seeds with MgSO_4_.

The salinity effects on germination and growth of cotton seedlings were investigated by 0.2% MgSO_4_-treated seeds in the laboratory. The emergence rate was determined on the 10th and 25th days after sowing. Then, on the 25th day after sowing, the germinated seedlings were collected for their above (stem and leaves) and underground (root) parts for fresh weight measurements. The mean comparisons of all the studied phenotypic traits after 0.2% MgSO_4_ treatment on cotton seeds were carried out through statistical analysis. They unraveled significant differences of MgSO_4_ treatments for 4 h from other treatments at 8 h, 12 h, and CK samples regarding the investigated traits (Figure 1). These differences laid the basis for further genomic analyses.

### 3.1. Transcriptome Profile of MgSO_4_ Treated Cotton Seedlings

The samples from three salt stress treatments were collected in triplicates on the 25th day of sowing. The number of raw reads obtained after RNA sequencing was about 610 million reads, filtered via removal of adaptors and ambiguous or low-quality reads. Consequently, approximately 596 million (97.73%) clean reads were obtained. On average, 7.42 Gb of clean bases were obtained after each seedling sample with Q20% of 97.80% and Q30 of 93.75%. The clean bases data had GC contents ranging between 45.03% and 47.59% (Table S1).

**Figure 2:**
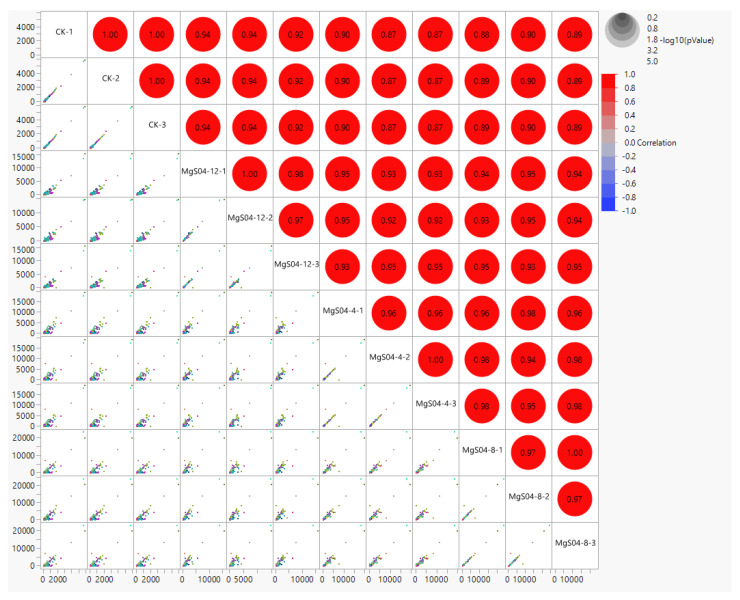
Correlation matrix of control and different MgSO_4_ treatments samples for 4, 8, and 12 h on cotton seedlings. CK: MgSO_4_ treatment at 0 h; M4: MgSO_4_ treatment at 4 h; MgSO_4_ treatment at 12 h, −1,2,3 representing replication.

After assembling, about 669,422,115 clean reads were aligned against the reference genome using the HISAT2 program. A set of 573,711,335 (86%) total mapped reads were generated. They comprised 59,774,675 (9%) secondary alignments and 513,936,660 (77%) unique alignments sited in the seeding tissues genes (Table S2). The FPKM values based on the gene expression level of 65,551 genes in samples from control and different levels of MgSO_4_ for 4, 8, and 12 h after sowing were demonstrated as the Pearson correlation coefficient graph depicting maximum significant positive relationships among four treatments (Figure 2, Tables S3–S8). These expressed genes were annotated for their functions through GO and KEGG classifications (Tables S9 and S10).

### 3.2. Identification of DEGs in CK and Treated Cotton Seedlings

For the prediction of candidate genes controlling the salt tolerance mechanism, the differentially expressed genes identified in pairwise comparisons (fold change) <1 down-regulated and >1 up-regulated ones in CK as compared with cotton seedling samples treated with MgSO_4_ for 4, 8, and 12 h. A total of 28,801 DEGs were discovered across CK and treatment comparisons of cotton seedling samples (Table S11).

**Figure 3:**
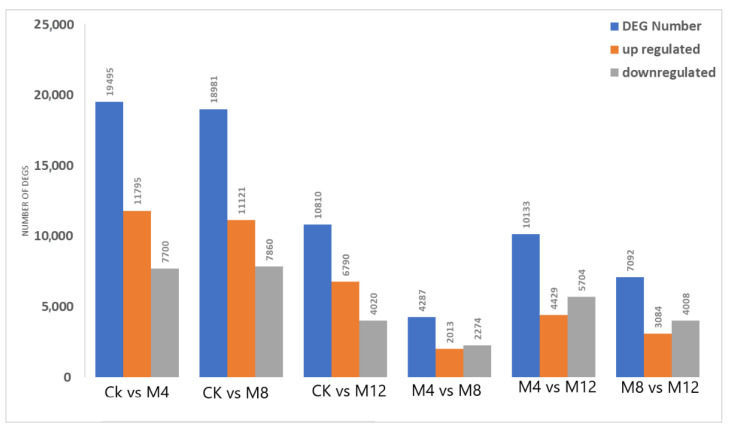
Summary of Differentially Expressed Genes (DEGs); X-axis displays all possible comparisons for MgSO_4_ treatments in differential expression patterns. Y-axis represented the number of DEGs as: blue colored bars showing total DEGs; Orange colored revealing up-regulated; and Gray colored depicting down-regulated DEGs. CK: MgSO_4_ treatment at 0 h; M4: MgSO_4_ treatment at 4 h; MgSO_4_ treatment at 12 h.

The identified DEGs with up-and downregulated expressions across CK and treatments comparison groups were as: CK-vs-M4: 19,495 (up—11,795; down—7700), CK-vs-M8: 18981 (up—11,121; down—7860), CK-vs-M12: 10,810 (up—6790; down—4020), M4-vs-M8: 4287 (up—2013; down—2274), M4-vs-M12: 10,133 (up—4429; down—5704), M8-vs-M12: 7092 (up—3084; down—4008) (Figure 3).

**Figure 4:**
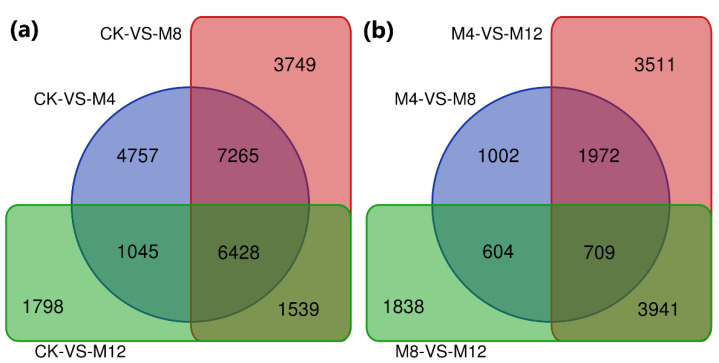
Venn diagrams illustrating DEGs. The sum of the numbers in each circle represents the total number of expressed genes within a comparison. In contrast, the numbers in the overlapping areas represent the number of expressed genes shared (**a**) among CK and treatment comparison groups and (**b**) among different treatment comparison groups of cotton seedlings. CK: MgSO_4_ treatment at 0 h; M4: MgSO_4_ treatment at 4 h; MgSO_4_ treatment at 12 h.

Further exploration of transcriptional changes among treated cotton seedling sample groups illustrated co-expression of 6428 DEGs among CK-vs-M4, CK-vs-M8, and CK-vs-M12 comparisons, 709 DEGs among M4-vs-M8, M4-vs-M12, and M8-vs-M12 comparisons. The maximum number of DEGs was estimated for their stable co-expression regarding salinity tolerance in the comparison groups CK-vs-M4 and CK-vs-M8 (Figure 4). These stably co-expressed DEGs in control and the treated seedling samples may have a main role in regulating salt sensory pathways, which require further exploratory studies regarding their roles in withstanding salinity in their growing mediums.

### 3.3. Functional Annotation of DEGs

For identified DEGs functions among CK, M4, M8, and M12 treatment groups seedlings, the annotated transcripts were explored for their functions related to salt stress tolerance. We discovered 4502 DEGs categorized through GO term classification as biological processes, cellular components, and molecular functions (Table S12). Among them, a prominent amount of DEGs concerning the biological processes category included response to salt stress, cellular response to salt stress, abscisic acid receptor PYR/PYL, regulation of seed growth and germination, auxin-activated signaling pathways, response to abscisic acid, gibberellic acid-mediated signaling pathways, and positive regulation of transcription elongation from RNA polymerase II promoter. Likewise, a significant count of DEGs with molecular functions related to the activation of DNA-binding transcription factors activity (GO:0019722), magnesium chelatase activity (GO:0016851), magnesium ion binding (GO:0000287), magnesium-dependent protein serine/threonine phosphatase activity (GO:0004722; GO:0004724), magnesium-importing ATPase activity (GO:0015444), regulation of ion transmembrane transporter activity (GO:0015095), regulation of seed germination (GO:0010029), positive regulation of response to salt stress (GO:1901002), cellular response to salt stress (GO:0071472), response to abscisic acid (GO:0009737), SNAP receptor activity (GO:0005484), and auxin-activated signaling pathways (GO:0009734) (Table S12).

**Figure 5:**
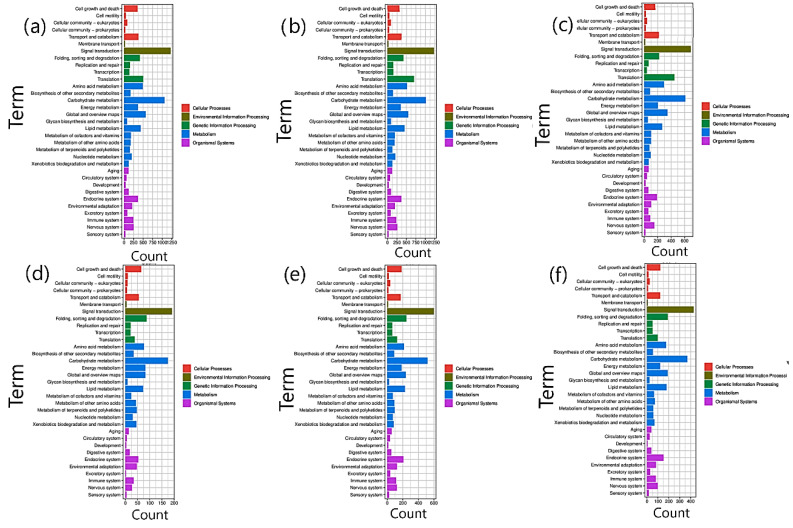
Heat map showing KEGG enrichment analysis of significant DEGs grouped into 15 classes involved in different functional pathways changed their expression significantly in the CK and 0.2% MgSO4-treated sample groups (**a**) CK_vs_M4, (**b**) CK_vs_M8, (**c**) CK_vs_M12, (**d**) M4_vs_M8, (**e**) M4_vs_M12, and (**f**) M8_vs_M12. The color gradient in this shape’s background reveals the corresponding *p*-value. Legends on the right are the description of the color gradient of *p*-value and classes of functional pathways.

The KEGG pathway analysis revealed 23,313 DEGs related to 127 significant KEGG pathways. The largest class observed was of ribosomes: ko03010 (1006), followed by plant hormone and signal transduction: ko04075 (898), carbon metabolism: ko01200 (689), starch and sucrose metabolism: ko00500 (648), biosynthesis of amino acids: ko01230 (616), and protein processing in the endoplasmic reticulum: ko04141 (607). These outcomes gave a perspective of activation of DEGs in seedling samples treated with 4 h of MgSO_4_ as the salt stress-related genes were expressed in those treated samples. The expression profiles revealed their significant roles in the hormones and signal transduction, carbohydrates metabolism, and biosynthesis of amino acids, promoting salt-stress tolerance (Figure 5, Table S10). Approximately 1818 transcription factors were observed in function annotations of the discovered DEGs. A more significant amount of ethylene-responsive transcription factors *ERF* (1235) was identified as differentially expressed, followed by *bHLH* (252), *WRKY* (96), *MYB* (202), *GATA* (81), *RABA* (64), *DIVARICATA* (28), *MADs*-*box* (26), and many others in the treatment seedling samples (Table S12).

### 3.4. Metabolome Profiling

**Figure 6:**
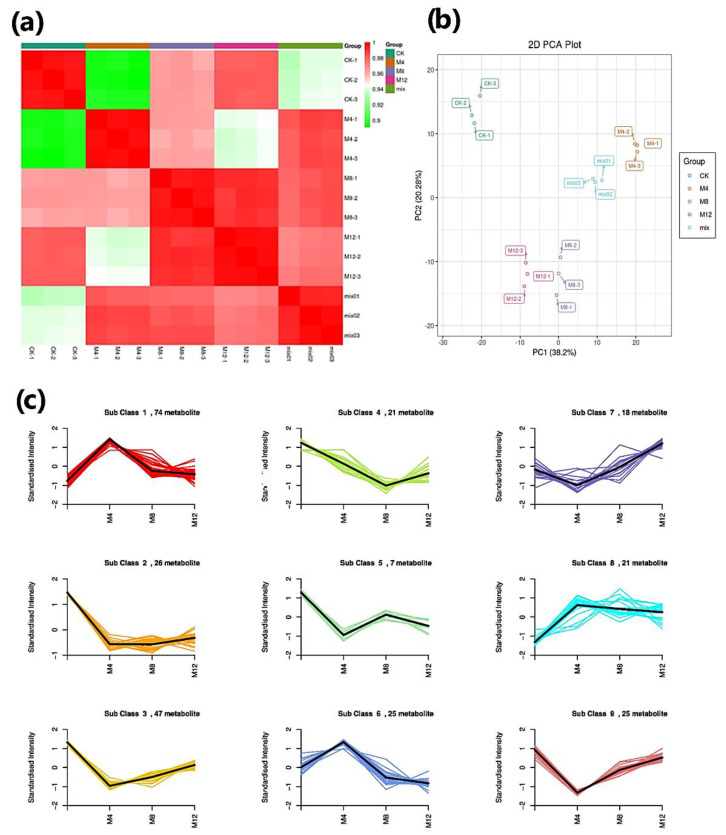
Quality control of metabolites identified in the experimental seedling sample extracts. (**a**) Pearson’s correlation coefficients; (**b**) Principal component analysis (PCA) of metabolites extract of cotton seedlings from CK and treated groups after 4, 8, and 12 h treatment with MgSO_4_; each sample in triplicates and quality control mix for metabolomics; (**c**) K-means diagram of the differentially accumulated metabolites among treated seedling sample groups (CK: MgSO_4_ treatment at 0 h; M4: MgSO_4_ treatment at 4 h; M8: MgSO_4_ treatment at 8 h; M12: MgSO_4_ treatment at 12 h; −1,2,3 representing replications). The x-axis represents the sample groups, and the Y-axis represents the relative content of standardized metabolites. Sub-class represents the number of the metabolite category with the same changing trend, and the metabolite represents the number of metabolites in the category (metabolites within each sub-class are given in Supplementary Table S13).

To better explore mechanisms or pathways underlying salt stress tolerance, the seedling samples were grouped into four (three treatments and one control), each with three biological replicates, for the qualitative and quantitative metabolite analyses. The correlation coefficients and PCA were determined to understand the differences between samples of treated groups, quality control (QC), and their variability size. These four seedling sample groups showed a trend of clear separation among them in score plots revealing differences in their metabolomes (Figure 6a). The first two PCs represented the maximum slope, covering a 38.2% variation under PC1 followed by 20.28% variation by PC2 with a cumulative variation 58.48% covered by these two PCs; hence, we created a biplot to represent it, as shown in Figure 6b. Different components covering individual and cumulative variation are shown in Figure S4 and different PC1 biplots covering variation are presented in Figure S5. The K-Means clustering revealed the detection of the metabolite from nine clusters to examine the metabolite’s relative content change in sample group comparisons. The metabolites in Sub-classes 1, 4, 6, and 8 exhibited their higher accumulation, such as 74, 21, 25, and 21 metabolites, in sample groups treated with MgSO_4_ for 4 h. All the sub-classes showed a standard intensity of more than one regarding metabolites accumulation (Figure 6c).

Further, DAMs were envisioned regarding their changes among comparison groups through the OPLS-DA model, where R^2^X, R^2^Y and Q^2^ values were around 0.7, 1, and 0.9, respectively, suggesting the reliability and stability of the model used. Metabolites with criteria of variable importance in projection (VIP) value ≥1 as well as top fold change (FC) ≤0.5 to ≥2 were taken as differential metabolites for the MgSO_4_-treated group discrimination from the CK group (Figure S1). A total of 264 metabolites were detected and divided into six groups of comparisons, based on the HCMC detection platform and self-built database (Table 1).

**Figure 7:**
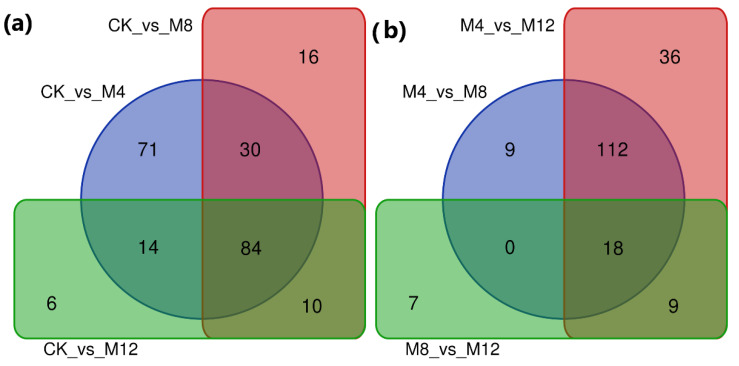
Venn diagrams for the representation of consensus results of DAMs classifying the samples by metabolites accumulated in treated samples (**a**) among CK and treatment comparison groups and (**b**) among different treatment comparison groups of cotton seedlings. CK: MgSO4 treatment at 0 h; M4: MgSO4 treatment at 4 h; MgSO4 treatment at 12 h.

Under different treatment conditions, the metabolites accumulated in the pericarp during the browning process were illustrated through a heatmap in cluster analysis. The core conserved DAMs co-expression found among control (CK) and three treatment comparison groups were 84 DAMs among CK-vs-M4, CK-vs-M8 and CK-vs-M12 comparisons as well as 18 DAMs between M4-vs-M8, M4-vs-M12, and M8-vs-M12 comparison groups. These DAMs may have major contributions in the regulatory pathways related to the salt-tolerance mechanism. A total of 71 DAMs were commonly differentially accumulated in the comparison group CK-vs-M4 and 36 in the M4-vs-M12 comparison group. These two comparison groups demonstrated a maximum number of DAMs due to having treatment of MgSO_4_ for 4 h common in them (Figure 7), illustrating the 4 h treatment time as best for enhancing salt tolerance of seeds.

**Figure 8:**
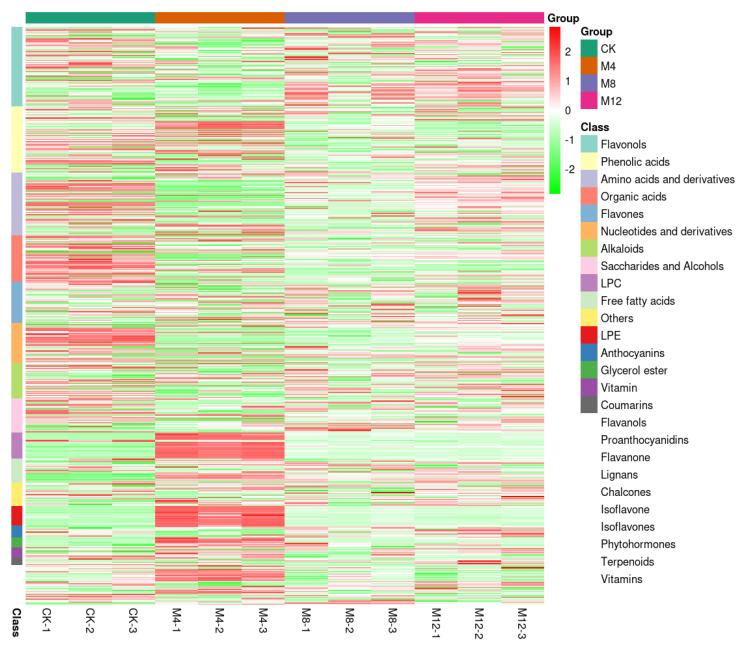
Heat map analysis of DAMs exhibiting fold change of top significant (*p* < 0.05) DAMs grouped into more 16 classes related to different treatment groups and CK. The four columns represent the treatment groups and CK samples (Green: Control (0 h), Brown: M4 (4 h), Purple: M8 (8 h), Magenta: M12 (12 h)) with further three sub-divisions, one for each biological replicate in every sample group. The correlation coefficients were utilized to classify different features determined by Pearson correlation based on average/means as a clustering algorithm. The color gradient from green (–2) to red (2) depicts the number of compounds, presented as relative fold change.

These DAMs from four cotton seedling sample groups were divided into more than 16 groups of flavonoids, phenolic acids, amino acids and derivatives, organic acids, flavones, nucleotides and derivatives, alkaloids, saccharides, and alcohols, LPC, free fatty acids, anthocyanins, glycerol ester, LPE, and others (Figure 8).

The KEGG enrichment terms related to DAMs were determined regarding comparison groups of CK, M4, M8, and M12. The KEGG classification based on significant metabolites with significant differences showed higher proportions of metabolites annotated to ‘metabolic pathways’ (84.3–93.8%) and ‘biosynthesis of secondary metabolites’ (29.4–44.2%) classes in all comparison groups viz CK-vs-M4, CK-vs-M8, CK-vs-M12, M4-vs-M8, M4-vs-M12, and M8-vs-M12 (Figure S2a–f; Table S14).

### 3.5. Conjoint Analysis

**Figure 9:**
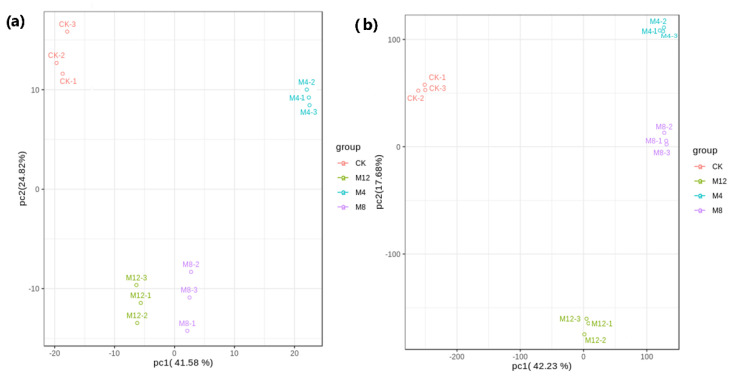
Comparison of principal component analysis of (**a**) DAMs and (**b**) DEGs associated with Ck and MgSO_4_ treatments in cotton seedlings. (CK: 0 h, M4: 4 h, M8: 8 h, M12: 12 h).

**Figure 10:**
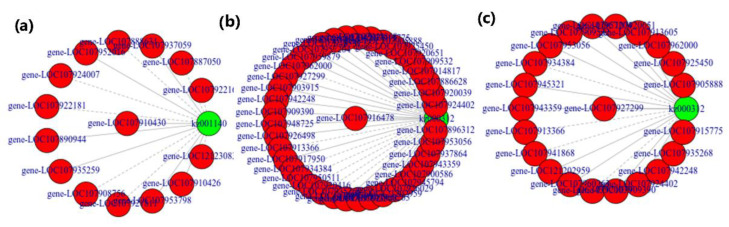
Correlation network diagram for expression of DEGs and DAMs abundance related to Glucose-1-phosphate, A-Ketoglutaric acid, and L-Glutamine under salt stress from treated seedling sample comparison groups: (**a**) CK-vs-M4, (**b**) CK-vs-M8, (**c**) CK-vs-M12, CK: MgSO_4_ treatment at 0 h; M4: MgSO_4_ treatment at 4 h; M8: MgSO_4_ treatment at 8 h; M12: MgSO_4_ treatment at 12 h. The green circles represent the regulatory pathways metabolites and red circles are for the representation of genes involved in the expression. The solid connecting line is positive and dotted for negative correlations.

Both transcriptome and metabolome data were integrated and statistically analyzed to examine the relationship between genes and metabolites at different levels and simultaneously coupled with other analyses such as PCA, correlation analysis, functional analysis, and metabolic pathways enrichment to screen out key genes’ metabolic pathways. Based on PCA scatterplots, the triplicated samples groups were separated and the samples from treatment M4 showed a distinct place from other treatment samples both in metabolites and transcriptome data results (Figure 9). According to this experiment’s differential metabolite analysis results, combined with the transcriptome differential gene analysis results, the same group’s differential genes and differential metabolites were simultaneously mapped through Cytoscape software to the KEGG pathway diagram to understand the relationship between genes and metabolites better (Figure 10). A total of 8018 DEGs were discovered in association (Pearson’s correlation coefficient ≥ 0.8) with 264 metabolites, with most of them jointly controlling the regulation of single or multiple metabolites. Most of the DEGs and associated metabolites in the interactive networks (Figure 10) showed involvement in the production and regulation of Glucose-1-phosphate, A-Ketoglutaric acid, and L-Glutamine under salt stress situations.

### 3.6. Verification through qRT-PCR

**Figure 11:**
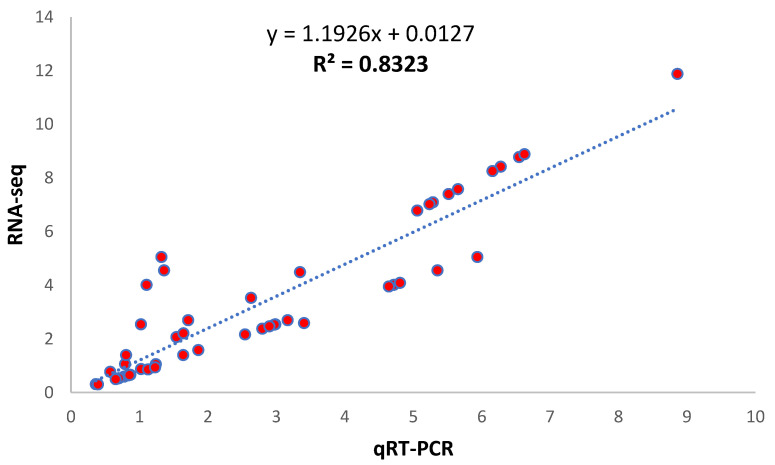
Validation of expression results using qRT-PCR representing the correlation between qRT-PCR and RNA-seq expression results for 10 selected DEGs related to salt-stress tolerance.

By integrating transcriptome data with metabolomics, 16 genes were considered to verify changes in their expression through qPCR (Figure 11; Table S15). There were genes related to Glucose-1-phosphate, A-Ketoglutaric acid, L-Glutamine, and transcription factors. A considerable amount of similarity was observed between transcriptome and qRT-PCR results, inferring the reliability of our reported results.

## 4. Discussion

The seedling stage in cotton is critical for growth and development due to higher sensitivity to biotic and abiotic stresses, especially salinity. Germination and seedling development are essentially required for good crop stand, which ultimately lead to high yield potential. It is crucial to explore the effects of different salts in the growing medium of cotton seedlings [54]. Salt, being a significant limiting factor for crop growth, yield, and production, is becoming a severe threat to most crop plants, and thus needs to be analyzed deeply. Salt stress induces other secondary stresses on plant-like osmotic, ionic, and oxidative stress [27,54]. Plants must overcome the adversities of salt stress by adjusting their physiological or biochemical processes [55]. Magnesium is centered in the chlorophyll molecule and is thus essential in photosynthesis’s normal conductance. Additionally, it plays several other significant roles in the plant life cycle such as plant respiration, activation of certain vital enzymes, phosphate metabolism, and protein synthesis. In the current study, the emergence percentage and root and shoot fresh weight illustrated marked differences among 0.2% MgSO_4_-treated samples for 4, 8, and 12 h. Particularly, a trend of better seedling growth was observed in the 4 h treated samples as compared with 8 and 12 h ones. The analysis of variance, PCA, and correlation analyses on phenotypic, transcriptomic, and metabolomic data revealed that there were significant differences among treatments and control and replications grouped together, which laid the foundation for further discovery of results. The transcriptomic results demonstrated an amount of 6428 and 709 core-conserved DEGs were shared commonly by CK and treated seedlings samples, inferring their significantly main roles in regulating salt tolerance. They need further attention and to be explored in detail regarding their exact role by determining the proteins/enzymes they code and when and how they switch on and off during growth in the saline mediums.

The salt tolerance mechanism is a complex quantitative trait controlled by several genes. Multiple studies have been conducted on cotton based on its salt tolerance ability by utilizing quantitative trait loci (QTL) by linkage mapping and genome-wide association studies. Little work on the transcriptomic and metabolomic aspects has been conducted yet [56,57,58]. Several QTL or genes were discovered by scientists in previous studies on the salt tolerance mechanism of cotton [55,59,60]. The salt stored in the soil depends on its type, as in sandy soil, there is a lesser amount for storage, but clayey soils store more of it. For Xinjiang, the area of salinized cultivated land accounts for 32.07% of the cultivated land area [61,62], and the annual loss of grain and cotton due to salinity and drought in the entire arid area will exceed hundreds of billions of yuan. Efforts into understanding cotton plant responses and adaptation mechanisms to severe salt stress conditions, such as the one from magnesium reported in the current study, are the key to improving cash crops to make them serve bio-saline agriculture. Plants developed high phenotypic plasticity, such as rapid responses to aggressive environmental factors and adaptations to changes [63].

A mature, dry seed starts germinating when it imbibes water, followed by radicle protrusion through rupturing the testa. It is a critical process as highly sensitive to imbalances in water, temperature, and oxygen concentrations and is regulated by different crucial phytohormones such as abscisic acid, gibberellins, auxin, cytokinins, ethylene and brassinosteroids [30,64]. Out of these, two highly significant phytohormones, i.e., GAs and ABA, work antagonistically, and are pivotal for the germination or dormancy of seed [65,66,67]. Besides, there are some signal molecules such as NO and ROS and external factors that affect germination significantly such as drought, temperature, salts, light, moisture, acidity, and nutrients [68]. Some phenylpropanoids like phenolic acids, flavonoids, coumarins, and monolignols [69] act as defensive agents in plants to combat biotic or abiotic stresses. Salicylic acid, a phenolic phytohormone, plays its role in the plant as a signaling molecule in response to diverse stresses [69].

The stress created by salts evokes osmotic pressure as well as toxicity in the environment of plants. The findings in the current study illustrated various changes in the genes, ultimate proteins, and metabolites after treatment with the 0.2% MgSO_4_. Generally, crop plants employed abscisic acid-dependent or -independent pathways to combat such stress and activate the downstream ABRE binding factor as a target [70]. In this study, the treated cotton plants utilized ABA-receptor PYL/PYR in the salt stress. They utilized the ABA signaling pathway in the seedlings treated for 4 h to control the osmotic stress by negatively regulating the abscisic acid signaling pathway via up-regulation of the protein-phosphatase (PP2C) [71]. It indicates that the seed treatment with the 0.2% MgSO_4_ for 4 h is the ideal time to enhance germination and growth of the seedling, as energy is saved from wastage/consumption by timely inhibition of the signaling transduction pathway.

Similarly, JA and SA played well-known roles against salt stress damage by working synergistically. The JA production is negatively regulated by the JAZ (jasmonate-associated ZIM domains) proteins [72] and positive regulation of the MYC2 protein to save energy [73]. The SA is renowned for its tolerance enhancement role under salinity conditions [74]. Moreover, the well-known TF “WRKY70” works downstream of *npr1* (nonexpressor of pathogenesis-related genes1) and is also involved in the SA-induced expression of pathogenesis related-1 (*PR*-1) genes. The CYTs are generally produced in the roots and then translocated to shoots via xylem tissues to promote growth and developmental processes, which are stopped under salt stress. The stoppage of CYTs to shoots alters the network of related gene expression [30,75]. The reduction of CYTs under salt stress could be a possible limiting factor for salinity tolerance enhancement [30,76]. Our conjoint analysis of DAMs and DEGs revealed higher expression of ‘Plant hormones signals transduction’ [77,78] at 4 h treatment time duration along with higher expression of JAZ proteins, which is proved to be the critical time point for enhancing the salt-tolerance mechanism in cotton seedlings. These findings are consistent with earlier findings on salt-stress studies on different crop plants [71,79].

## 5. Conclusions

In this study, 0.2% MgSO_4_ was applied to cotton seedlings for 4, 8, and 12 h to investigate plant salt tolerance regarding germination and seedling establishment. The study was carried out with the assumption of the potential reduction in cotton seedlings’ germination and seedling development as effects of salt stress, but positive effects of MgSO_4_ 4 h treatments were observed on germination and seedling establishment. It seems that Mg^+2^ impacted growth and germination by activating certain growth-promoting enzymes and metabolites in salt-treated seeds. These observations were also validated by transcriptomic and metabolomic findings revealing regulation of different growth-prompting hormone signaling pathways. These resultant findings revealed 4 h MgSO_4_ treatment as beneficial to alleviate adverse effects of salt stress in cotton. This pretreatment of MgSO_4_ on cotton seeds can be used in future breeding programs to enhance cotton growth and development with good crop stand under salinity stress.

## Figures and Tables

**Table 1:** Summary of significant differentially accumulated metabolites (DAMs) detected across CK and MgSO_4_ treatments (4 h, 8 h, and 12 h) comparison groups of Cotton seedling samples.

Group Name	All Sig Diff	Down-Regulated	Up-Regulated
CK_vs_M12	114	67	47
CK_vs_M4	199	104	95
CK_vs_M8	140	99	41
M4_vs_M12	175	89	86
M4_vs_M8	139	84	55
M8_vs_M12	34	6	28

CK: MgSO_4_ treatment at 0 h; M4: MgSO_4_ treatment at 4 h; M8: MgSO_4_ treatment at 8 h; M12: MgSO_4_ treatment at 12 h.

## Data Availability

Data supporting reported results is available as Supplementary Files.

## Supplementary Materials

The following supporting information can be downloaded at: https://www.mdpi.com/article/10.3390/life12060921/s1.
