# Supplementary figures and images for: Transcriptome and Metabolome Analysis of Upland Cotton (Gossypium hirsutum) Seed Pretreatment with MgSO4 in Response to Salinity Stress
Source: Life (Basel). 2022 Jun 20;12(6):921. doi: 10.3390/life12060921 (PMC9227556; doi:10.3390/life12060921)

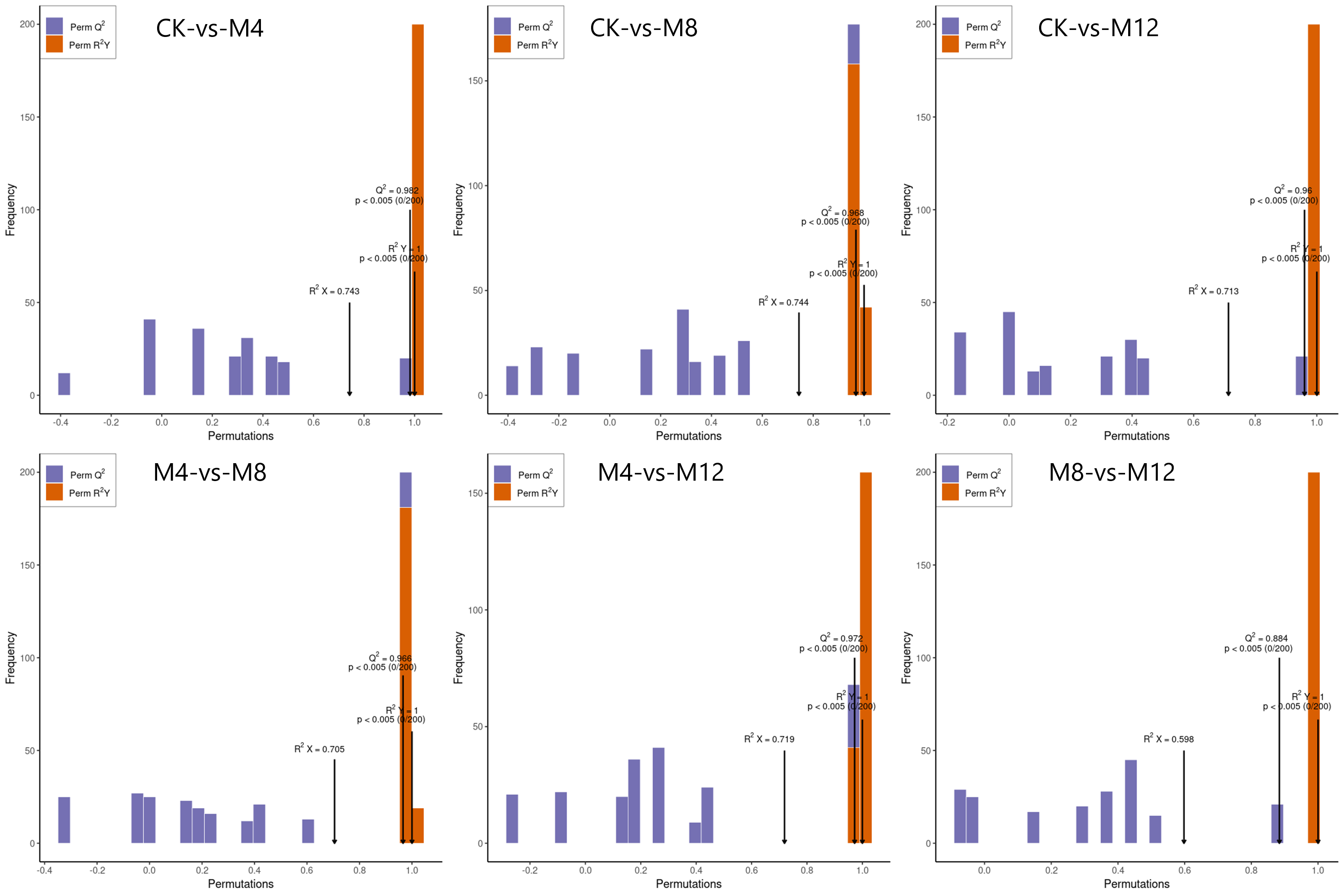

Supplement: Supplementary file 1 [file life-12-00921-s001.zip › Figure S1.tif]

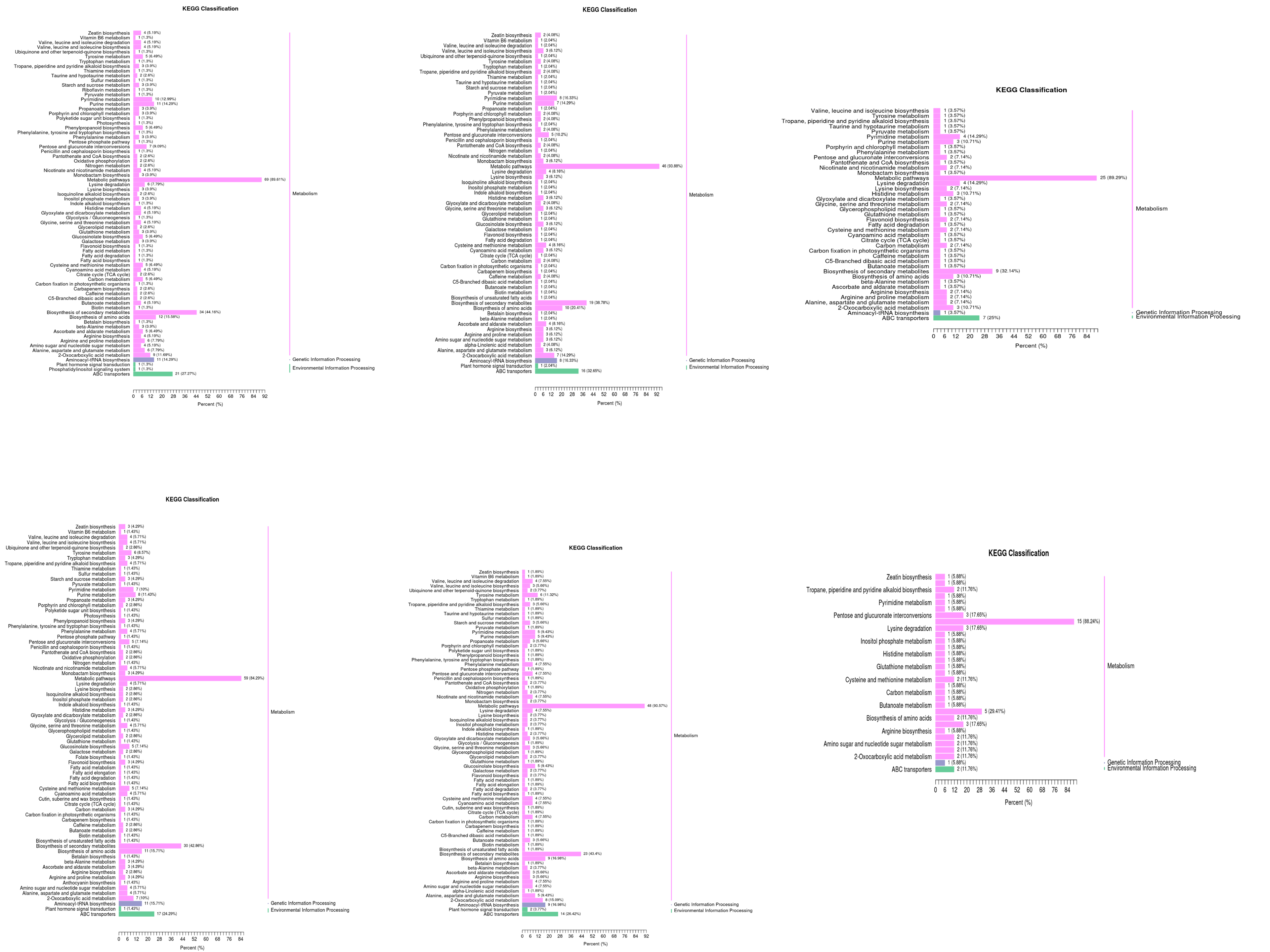

Supplement: Supplementary file 1 [file life-12-00921-s001.zip › Figure S2.tif]

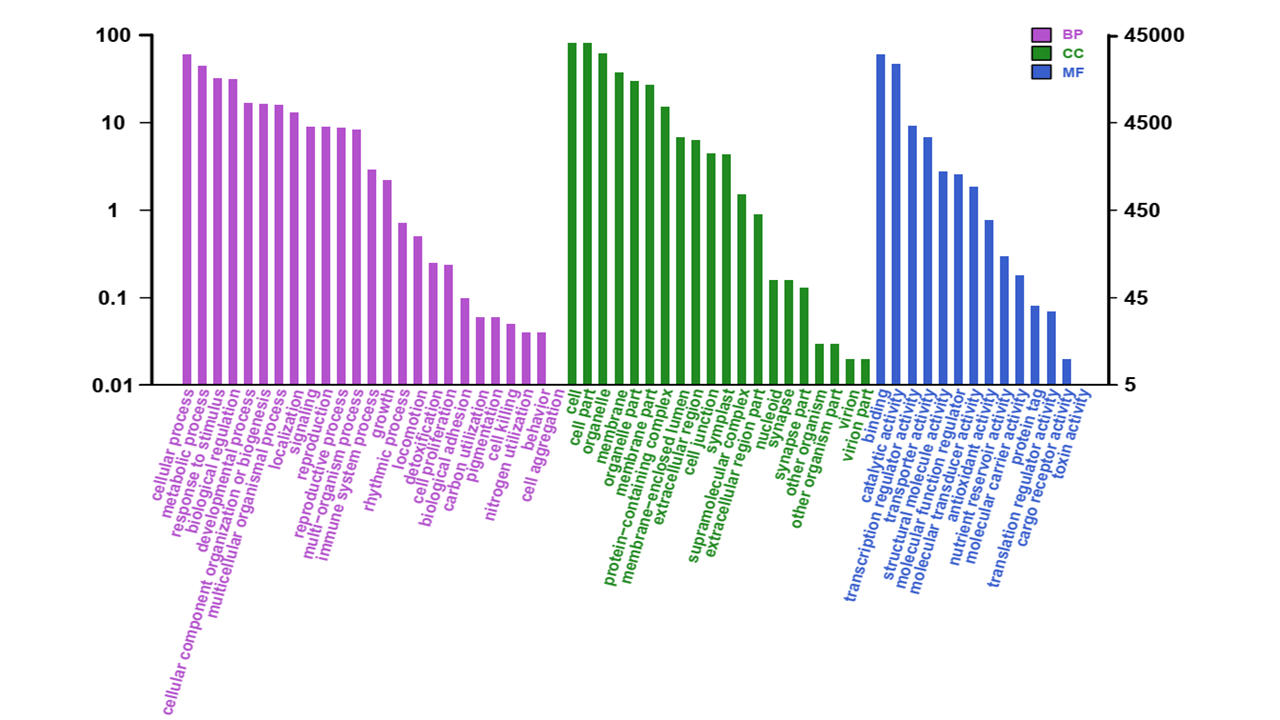

Supplement: Supplementary file 1 [file life-12-00921-s001.zip › Figure S3.tif]

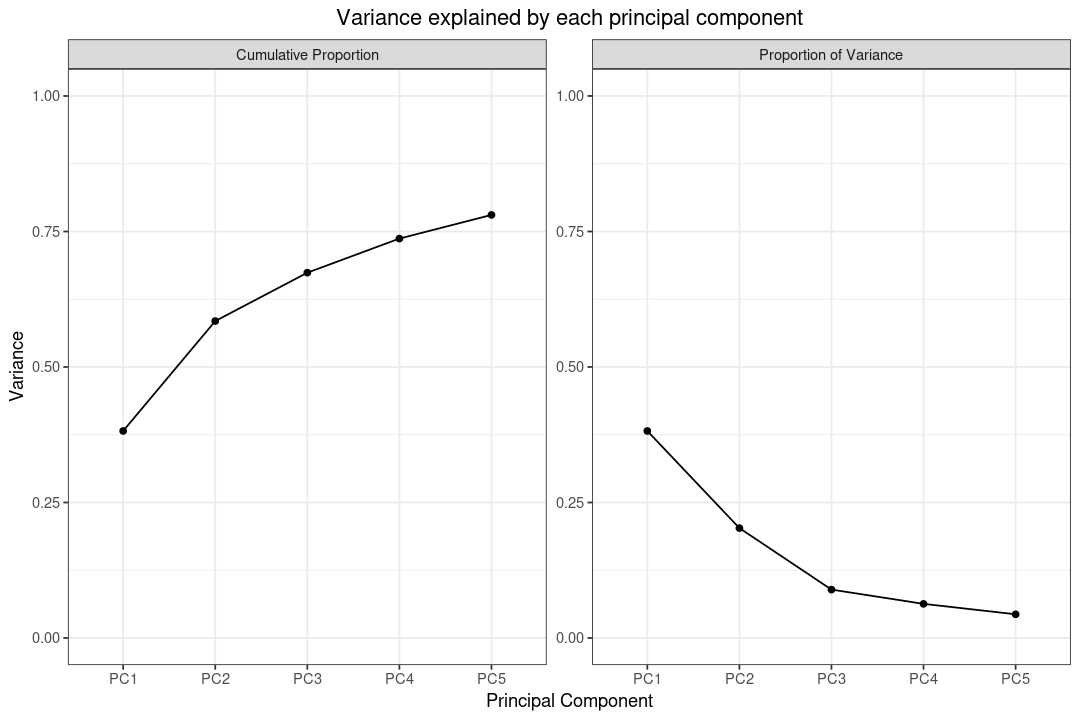

Supplement: Supplementary file 1 [file life-12-00921-s001.zip › Figure S4.png]

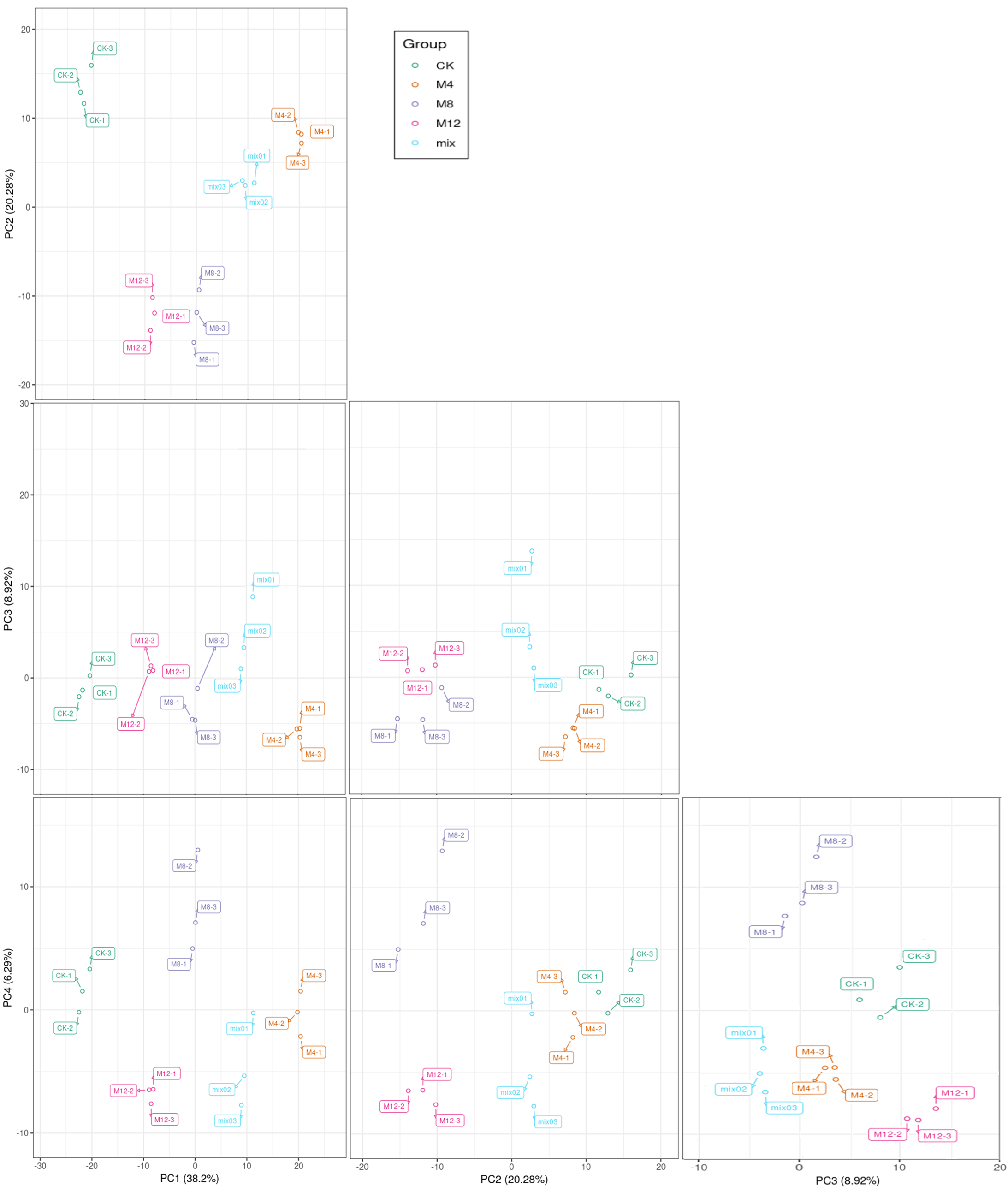

Supplement: Supplementary file 1 [file life-12-00921-s001.zip › Figure S5.tiff]
